# Microglia and Inflammatory Responses in Diabetic Retinopathy

**DOI:** 10.3389/fimmu.2020.564077

**Published:** 2020-11-06

**Authors:** Urbanus Muthai Kinuthia, Anne Wolf, Thomas Langmann

**Affiliations:** ^1^Laboratory for Experimental Immunology of the Eye, Department of Ophthalmology, Faculty of Medicine and University Hospital Cologne, University of Cologne, Cologne, Germany; ^2^Center for Molecular Medicine, University of Cologne, Cologne, Germany

**Keywords:** diabetic retinopathy, microglia, inflammation, cytokines, chemokines, hyperglycemia

## Abstract

Diabetic retinopathy is a vision-threatening disease affecting neurons and microvasculature of the retina. The development of this disease is associated with the action of inflammatory factors that are connected to the activation of microglial cells, the resident tissue macrophages of the CNS. In the quiescent state, microglial cells help maintain tissue homeostasis in the retina through phagocytosis and control of low-grade inflammation. However, prolonged tissue stress due to hyperglycemia primes microglia to become overly reactive with the concomitant production of pro-inflammatory cytokines and chemokines causing chronic inflammation. In this review, we provide evidence of microglial cell activation and pro-inflammatory molecules associated with the development and progression of diabetic retinopathy. We further highlight innovative animal models that can mimic the disease in humans and discuss strategies in modulating microglial-mediated inflammation as potential therapeutic approaches in managing the disease.

## Introduction

Diabetic Retinopathy (DR) is a disease that affects the neurons and microvasculature of the retina leading to vision deficits in people suffering from diabetes mellitus (DM). DR is clinically divided into two broad forms based on the severity of the disease; an early non-proliferative form (NPDR) and an advanced proliferative (PDR) form. NPDR is characterized by increased vascular permeability, capillary occlusion, retinal hemorrhages, hard exudates, thickening of the basement membrane, and pericyte drop-out in the retinal vessels. In contrast, PDR entails pathological neoangiogenesis, vitreous hemorrhage and retinal scars and detachment (1). A critical pathological hallmark of both NPDR and PDR is diabetic macula edema (DME), caused by alterations of the blood-retinal-barrier (BRB) and results in the vision loss in DR patients (2). In recent years, the administration of intravitreal anti-vascular endothelial growth factor (VEGF) agents such as aflibercept, ranibizumab, and bevacizumab has been regarded as a promising treatment for DR and DME (3). Unfortunately, treatment with anti-VEGF therapies is insufficient as nearly half of the patients fail to show significant clinical improvement (2).

DR was initially regarded as a microvasculopathy owing to the abnormalities in the retinal microvasculature such as microaneurysms and acellular capillaries (4). However, sufficient evidence has demonstrated that inflammation accompanies the progression of DR (5–7). This discovery has prompted the re-evaluation of the triggers for DR development and novel approaches are now focusing on the role of inflammation and immune system dysregulation in the pathogenesis of DR. The inflammatory reactions that occur locally in the retina are mediated by activated microglia which inhabit the plexiform layers of the retina (8). Upon encountering danger signals, microglia transform from a surveillance state to an activated state specialized to neutralize the noxious stimulus and restore tissue homeostasis (9). However, prolonged tissue stress primes microglia to become overly reactive and with a secretory phenotype associated with pro-inflammatory mediators including cytokines and chemokines (10). Therefore, microglia present a critical target for therapeutic intervention in the prevention and treatment of neurodegenerative diseases in the retina (10). Thus, in this review, we discuss the role of microglia and mediators of inflammation in DR pathologies and discuss developments in DR mouse models that can recapitulate the disease in humans. We will also highlight recent advances in regressing microglial inflammatory responses in the diabetic retina.

## Origin and Physiological Role of Microglia

Microglia are mononuclear phagocytes and can be regarded as the tissue-resident macrophages of the retina. There has been a debate regarding their origin but it is now generally accepted that they originate from the embryonic yolk sac and are self-renewing (11). The yolk sac derivation and preservation until adulthood suggest that microglia play an essential physiological role in the development of the CNS (12). Microglia can adapt to different regions of the CNS, where they exhibit distinct surface markers, density, and morphology in the areas they occupy. Indeed, microglia gene expression varies greatly by regions of the brain tissue (13) and they display broad functional states (14). Recent studies using single-cell RNA sequencing of brain microglia identified nine transcriptionally distinct microglial states which expressed unique markers (15). Similarly, molecular profiling of retinal microglia across different developmental stages uncovered the presence of unique cell clusters suggesting discrete transcriptional states during development (16). In the retina, microglia have been found to occupy different regions and with different functions thus confirming their phenotypic heterogeneity in homeostasis and disease (17). Although both retinal and brain microglia share a common origin and keep these tissues under surveillance, it is yet to be resolved whether the clusters in the brain and the retina are transcriptionally similar or different.

In early stages of retinal development, microglia inhabit the ganglion cell layer of the retina and are involved in phagocytosis of dying neurons (10). In the developed retinal tissue, ramified microglia are confined to the inner and outer plexiform layers from where they survey the retinal environment with their long processes (10), a phenomenon observed with brain microglia (18). Microglia cells in the inner plexiform layer are IL-34 dependent while those occupying the outer plexiform layer are IL-34 independent and play different functions during homeostasis but they relocate to the subretinal space (SRS) during neurodegeneration (17). As retinal tissue macrophages, they represent about 5%–12% of the CNS cell population and display a unique genetic signature by the expression of transmembrane protein 119 (Tmem119) and purinergic receptor P2ry12 (19, 20).

These glial cells not only possess the protective ability but also play a part in the development and preservation of neuronal circuits. In healthy situations, microglia through their receptors for neurotransmitters establish molecular links with neurons and other cells through which they participate in the maintenance of neural networks and the removal of cell fragments through phagocytosis (21–23). It is worth to mention that microglia detect specific stimuli in the neuronal surroundings by its sensome which includes an array of surface molecules among them receptors for immune components such as cytokines, neurotrophic factors, antibodies, complement, and adhesion molecules (10, 24). Some of these receptors such as CD200R which is a receptor for the transmembrane glycoprotein CD200, once activated triggers cellular signaling aimed at the maintenance of tissue homeostasis by modulating microglial activation in the retina. Also, healthy neurons release fractalkine (CX3CL1), a chemokine that binds to its receptor (CX3CR1) on microglia cells and helps to modulate their reactivity (25, 26).

Additionally, the CX3CL1-CX3CR1 signaling regulates microglia repopulation thereby restoring their physiological function and maintenance of retinal homeostasis (27). The expression of microglial cell surface markers varies across developmental stages and disease states and it is difficult to pinpoint exact surface markers for microglia. For instance, during development microglia express markers including CD11b, CD45, CD68, F4/80, and isolectin which are also displayed by monocyte derived macrophages making it difficult to discriminate CNS microglia from other macrophages. However, a distinction between resident microglia and infiltrated macrophages can be partially resolved in a time-dependent manner. That is because during inflammation in the CNS, microglia activation occurs within 1 day and precedes the detection of peripherally-derived macrophages infiltration which takes some days (28). Fate mapping techniques coupled to flow cytometry revealed that retinal microglia have a unique CD45^lo^, CD11c^lo^, F4/80^lo^ signature which is conserved in steady-state and disease while monocyte-derived macrophages express the same markers in higher quantities (29). While these discoveries shed more light in resolving the conundrum facing surface markers, it has been revealed that during pathological conditions CNS-infiltrating macrophages may acquire microglia-specific markers through reprogramming by the CNS niche (30). This phenotypic convergence between microglia and CNS-infiltrating macrophages results in functional similarities during disease progression.

## Microglia Phenotypes

Microglia cells display two main activation phenotypes owing to the intracellular signals within their surroundings (31–33). These pro-inflammatory and anti-inflammatory states have been also described as M1 and M2 respectively. However, we prefer here using the broader pro- and anti-inflammatory nomenclature. Inflammation is principally an immune response meant to protect tissues from harm (8, 34). When cell surface receptors detect damage-associated molecular patterns (DAMPs), microglia change their phenotype from a highly ramified form to an amoeboid shape with retracted processes and high motility (9). The classical pro-inflammatory form exhibits a secretory phenotype usually characterized by pro-inflammatory cytokines (IL-1β, IL-6, IL-8, TNF-α). This type of inflammation is beneficial to the retinal environment and once danger molecules are eliminated, microglia downregulate the pro-inflammatory response and revert to the state of surveillance. Alternatively, microglia can shift to an anti-inflammatory phenotype, also referred to as alternatively-activated microglia and secrete anti-inflammatory cytokines including IL-4, IL-10, IL-13, transforming growth factor (TGF-β) and arginase 1 and promote clearance of debris to maintain homeostasis (31). The pro-inflammatory phenotype displays specific surface receptors including CD16 and CD32 whereas the anti-inflammatory phenotype is characterized by markers such as CD163 and CD206 (35, 36). The homeostatic control of the retinal tissue by microglial cells emanates from their complex immune checkpoints and plasticity, a unique phenomenon of these sentinels of the innate immune system. However, in some instances, especially during chronic disease, inflammation may fail to resolve due to continued tissue stress and danger signals which trigger a positive feedback mechanism of the pro-inflammatory component. In this state, the immune checkpoints of microglia become counterproductive and block the ability to restrain inflammation (37). The excessive release of pro-inflammatory molecules by microglia is deleterious and worsens the disease pathology. During diabetes, cytokine-mediated inflammatory responses are highly manifested in the diabetic retina and this results in increased proliferation of microglia which shift from the anti-inflammatory to the pro-inflammatory state and aggravate inflammation. Apart from microglia, diabetes induces the secretion of vascular endothelial growth factor (VEGF) and TNF-α by Müller glia which further aggravates the progression of DR (38).

Recent studies have demonstrated that microglia are capable of displaying activation states which can neither be classified as pro- nor as anti-inflammatory. Transcriptomic analysis of immune cells in brain pathologies has identified a phenotype of microglia termed as disease-associated microglia (DAMs) (39, 40). DAMs may recognize, contain and remove neurodegeneration-associated molecular patterns (NAMPs) present in CNS pathologies (39, 41). This microglia phenotype is conserved in both mice and humans implying that it plays a pivotal role in disease progression in the CNS (39). Other than DAMs found in the brains of several models of Alzheimer’s disease, a unique heterogenous population of monocyte derived cells known as the border associated macrophages (BAMs) has been found at the brain’s border regions (42). BAMs in the brain display a transcriptional profile similar to DAMs suggesting an immune diversity in the brain (42). The retinal borders, just like the brain, are associated with a limited population of perivascular macrophages (PVMs) which are found close to capillary walls (43). A common feature of both the BAMs and PVMs is that they are embryonically derived and long-lived. Differences between resident microglia and PVMs can be partially observed in the surface markers. Thus, resident microglia are characterized by a CD206^low^/CD163^-^ signature while PVMs display a CD206^high^/CD163^+^ signature (35). During breakdown of the blood-retinal barrier (BRB) and retinal degeneration, PVMs migrate to the site of injury (43) where they together with activated microglia aggravate disease pathology (44). In virus infected brains, microglia can express additional unique markers including P2ry12 and Fc receptor-like S (Fcrls) (45).

## Morphological Analysis of Microglia in Animal Models and Patients of DR

Several animal models have been used to study the activation of microglia in diabetes and have confirmed the phenotypic changes mentioned herein. In the streptozotocin (STZ)-induced diabetic rat, microglia change their morphology from a ramified to an amoeboid form and increase in number within the outer plexiform layers and outer photoreceptor layer suggesting that activated microglia are highly proliferative and migratory (46). In another study involving STZ rats, the number of activated microglia increased progressively in a time point manner but there was no difference in microglia density when compared with the control animals (47). In C57BL/6 mice treated with alloxan followed by daily administration of insulin, microglia cells with shortened dendrites and enlarged soma were observed without apoptosis of retinal neurons, suggesting that microglial reactivity ensues early in progression to DR (48). In the db/db mouse model of type 2 diabetes, activated retinal microglia were found in the inner nuclear layer and switched from an anti-inflammatory phenotype during early disease towards a pro-inflammatory phenotype in late stage (49). Chronic hyperglycemia in the Goto Kakizaki (GK) rat induced microglial proliferation, trafficking, and accumulation in the subretinal space (50). A notable observation in the GK diabetic rat is that as the number of pores in the retinal pigment epithelium (RPE) cells decreased there was an increased proliferation of microglia in the SRS. This transcellular pathway was active during early stages of diabetes (5 weeks) but the pores decreased during late stages of diabetes (12 weeks) which resulted in the subretinal accumulation of activated microglia.

In the mouse model of oxygen-induced retinopathy (OIR), resident microglia with a proliferative and migratory phenotype were found to occupy ischemic and neovascular regions of the retina (51). Further studies using retinal cryosections of OIR mice revealed that amoeboid microglia were present in the superficial and nerve fibre layers and were associated with neovascularization (52). Similarly, clinical studies have confirmed microglia activation in the retina of both NPDR and PDR patients whereby, in NPDR microglia migrated into the plexiform layers and proliferated whereas, in PDR, microglia soma were highly hypertrophied and assembled around ischemic regions (53). Our understanding of microglial phenotypic changes has been hampered by the lack of studies in animal models that clearly mimic the NPDR and PDR in humans. Further, hyperreflective spots (discrete microaggregates) were observed in the inner retinal layers of DR patients and later in the outer retinal layer indicating that they may correspond with the progression of retinopathy (54). The hyperreflective spots are thought to be aggregates of activated microglia (54) and their presence in early DR suggests that a resident immune cell response occurs before late DR.

### Effects of Glucose Fluctuation on Microglia—*In Vitro* Studies

Elevated glucose levels and glucose fluctuations can induce microglia polarization in cell culture systems. The treatment of rat microglia with increasing glucose concentrations upregulated expression of TNF-α and monocyte chemotactic protein-1 (MCP-1, alias CCL2) in a time dependent manner (55). A study using the BV-2 cell line showed that a shift from normal glucose (5mM) to high glucose (25 mM) enhanced microglia proliferation, secretion of pro-inflammatory factors (TNF-α and oxygen radicals) and stress proteins including heat shock protein 70 (HSP70), heme oxygenase 1 (HO-1) and inducible nitric oxide synthase (iNOS) (56). In a separate study, high glucose levels increased the production of IL-1β in retinal cell culture, which induced microglia cell proliferation (57). As microglia cells continue to proliferate in the high glucose environment, they may reprogram their checkpoints and respond by secreting more inflammatory factors. The maintenance of cytokine production by proinflammatory microglia is dependent on glycolysis and glucose transporter 1 (GLUT1) has been found to regulate this process (58).

## Mediators of Inflammation in Diabetic Retinopathy

**Figure 1:**
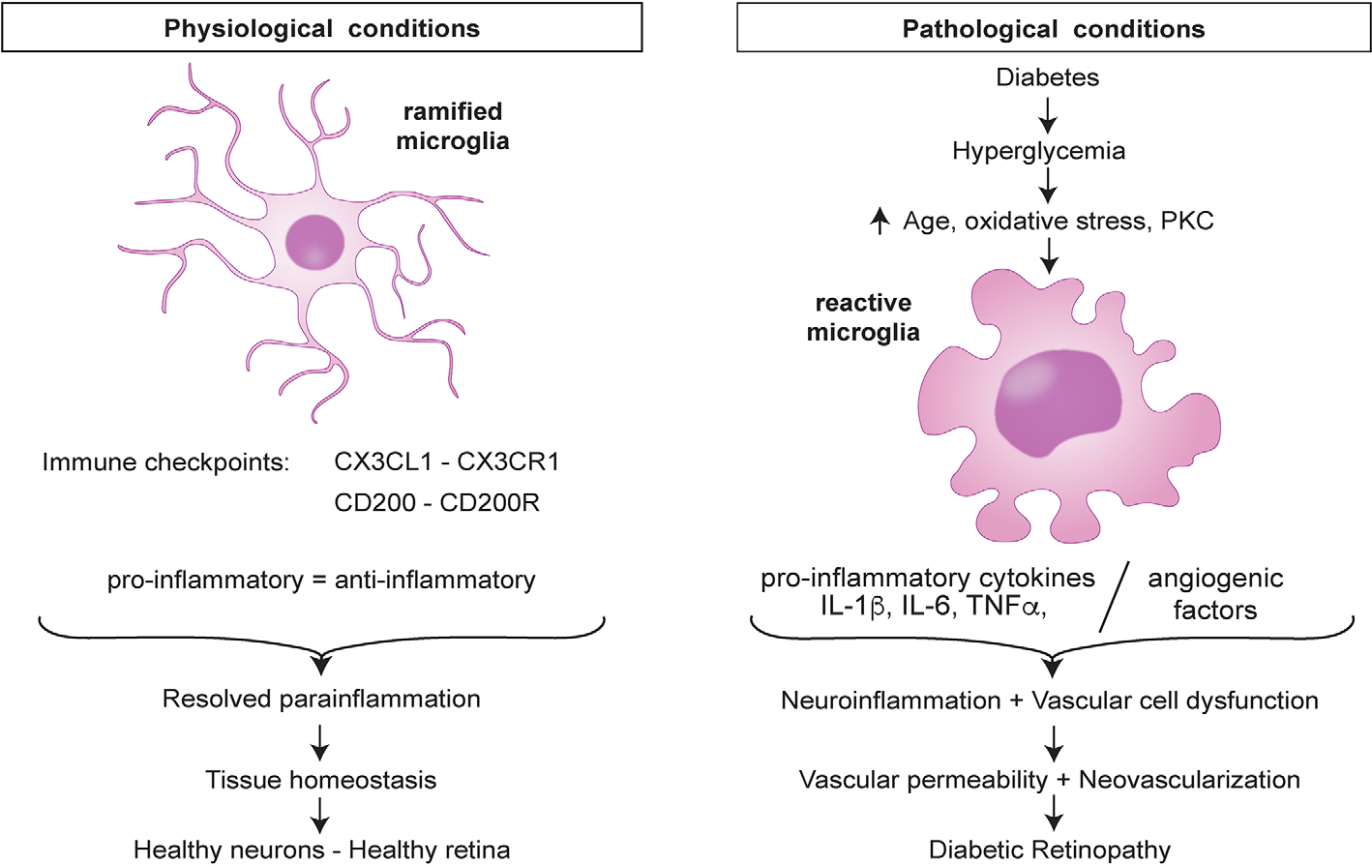
Retinal microglia dysregulation in diabetic retinopathy.

At the onset of DR, the BRB offers a physical barrier that prevents circulating cells from entering the retina and this leaves microglia and other retinal cells as the only immune sentinels of the retina. In this state, the low-level tissue insults are detected by microglia through their pattern recognition receptors (PRRs). Microglia maintain several immune checkpoints to restore tissue homeostasis. In the presence of an early noxious stimuli such as hyperglycemia, microglia maintain a balance of the pro- and anti-inflammatory states but as the tissue insults persist the balance is lost and the cells are reprogrammed to attain a new threshold characterized by the pro-inflammatory state which is harmful (**Figure 1**).

Hyperglycemia, a key feature of DM leads to elevated production of superoxides in mitochondria and enhances the flux of polyol hexosamine and protein kinase C (PKC) pathways. This phenomenon results in increased production of reactive oxygen species (ROS) and accumulation of advanced glycation or lipoxidation end-products (AGE or ALEs) which affect retinal physiology (59). Interactions between AGE and AGE receptor (RAGE) can induce the proinflammatory phenotype in microglia which results in increased secretion of inflammatory cytokines (TNF-α, IL-6) (60, 61). Moreover, AGE-RAGE interactions activate signaling pathways that result in endothelial permeability and amplification of cytokines and proangiogenic factors. AGE can induce the secretion of CCL2 by neuronal cells which stimulates the expression of TNF-α in microglia *via* extracellular signal regulated kinase (ERK) and nuclear factor kappa B (NF-kB) (62). The hyperglycemic microenvironment also leads to increased glycolysis by microglia and subsequent production of ATP and electrons *via* the mitochondrial electron transfer chain. The free electrons bind to oxygen molecules to form ROS. Further, ROS and oxidative stress can induce an inflammatory response in the retina through nuclear translocation of NF-kB and expression of TLR-2 and TLR-4 (63, 64), thereby upregulating inflammatory responses in retinal microglia. Hyperglycemia-initiated electron transport chain dysfunction has been linked to electron leakage through complex I and III leading to increased ROS and superoxide levels (65). This glucose-induced production of superoxides can activate the nuclear translocation of NF-kB which modulates the expression of genes encoding pro-inflammatory cytokines (IL-1β, IL-6, IL-8, TNF-α, VCAM-1, and ICAM-1) and chemokines (CCL2, CCL5, CCL12) (66, 67).

Hyperglycemia can also activate VEGF expression, hypoxia-inducible factor-1 (HIF-1) translocation to the nucleus and ERK1/2-NF-kB signaling pathway in microglia (68, 69). Overexpression of VEGF contributes to retinal neoangiogenesis, whereas the translocation of HIF-1 activates transcription of genes involved in angiogenesis. Additionally, microglia activation and secretion of CCL2, have been implicated in the formation of subretinal neovascular tufts in the retina of *Vldlr^–/–^* mice (70). Considering the proliferative stage of DR, recent studies have shown that microglia are hyperglycolytic during pathological angiogenesis in the OIR model (71). The glycolytic pathway leads to increased production of acetyl coenzyme A which induces histone acetylation and reprogramming of microglia to an angiogenic phenotype supporting neovascularization (71). Increased levels of TNF-α and IL-1β coincide with increased intraretinal neovascularization in DR and microvascular degeneration in ischemic retinopathy (72, 73). Microglia can also enhance vascular alterations in the retina of Goto Kakizaki rats of pharmacologically induced diabetes (74). Taken together, these studies add to the growing knowledge that hyperglycemia induces a cascade of cellular events coordinated by microglia and drive the pathophysiology associated with DR.

The initial inflammatory response of microglia is corrective and involves the release of anti-inflammatory cytokines including IL-4, IL-10, IL-13, and transforming growth factor-beta (TGF-β) that resolve inflammation and enhance survival of neurons (75). However, sustained hyperglycemia and other tissue insults trigger the overproduction of the pro-inflammatory factors by already neurotoxic microglia. The over-reactive microglia secrete large proportions of pro-inflammatory and cytotoxic molecules such as TNF-α, IL-1β, ROS, and reactive nitrogen species (RNS) (76) which cause chronic inflammation leading to damage of the BRB in the diabetic eye and worsening of the pathology of DR. The secretion of inflammatory molecules leads to activation of other glial cells such as astrocytes which respond by amplifying neuroinflammation. Clinical evidence has proven that genes encoding several inflammatory mediators are highly expressed in the vitreous of proliferative DR patients. The immune repertoire of inflammatory molecules found in the diabetic eye and serum of DR patients from studies involving healthy controls are summarized in **Table 1** below. Further studies by Yoshimura and colleagues revealed that a strong positive correlation exists between IL-6, IL-8, and CCL2 expression levels (82). Other findings have established that elevated levels of VEGF, IL-6, CCL2, IL-8, inducible protein-10 (IP-10), and hepatocyte growth factor (HGF) in the aqueous humor were associated with the presence of DR (83, 86). It has also been shown that plasma levels of S100A12 are associated with the progression of DR (87). S100A12 is a calcium binding protein that can induce microglia activation and inflammation in diabetic rats (88). In view of the plethora of similar pro-inflammatory cytokines and chemokines found in samples from diabetic patients, it is evident that hyperglycemia plays a crucial role in triggering microglia cell activation and the molecular events that lead to the release of mediators of inflammation in DR.

**Table 1:** Inflammatory molecules (cytokines, chemokines, angiogenic factors) upregulated in conditions of diabetic retinopathy.

**Analyzed tissue**	**Study cohort (n)**	**Inflammatory mediators**	**Reference**
Vitreous humor	60	IL-6, IL-8, MCP-1, VEGF	(77)
Vitreous humor	82	IL-1β, IL6, IL8, CCL2, EDN1, VEGF, TNF-α	(78)
Vitreous humor & serum	68	IL-6, IL-8, TNF-α	(79)
Vitreous humor	80	IL-1β, IL-6, IL-8, CXCL-9, CXCL-10, TNF-α	(80)
Vitreous humor	160	IL-6, IL-8, IL-13, IP-10, MCP-1, MIP-1β, PDGF, VEGF	(81)
Vitreous humor	21	IL-1β, TNF-α	(6)
Vitreous humor	50	IL-1β, TNF-α, IL-6, IL-8	(5)
Vitreous humor	345	IL-6, IL-8, MCP-1,	(82)
Aqueous humor	61	IL-1β, IL-2, IL-4, IL-5, IL-6, TNF-α, VEGF	(83)
Retinas of rats & human retinal endothelial cells	N/A	CCL2	(84)
Retinal endothelial cells & vitreous humor	69	CCL2, CCL-4, CXCL-9, CXCL-10, MCP-1	(85)

Microglia also produce complement proteins and regulators which contribute to retinal homeostasis and disease (89, 90). While microglia are known to express C1q, cytokines (IL-1*β*, IL-6, and TNF-*α*) can induce the secretion of C1r, C1s, and C3 by astrocytes and microglia (91). Inflammation at the onset of DR can activate the complement system as an internal mechanism to restore homeostasis (9, 44). However, chronic inflammation due to sustained diabetic insults dysregulates the complement system through production of AGEs which associate with mannose binding lectin (MBL) to disturb retinal physiology (92). Separately, C1q has been found to propel microglial activation and neurodegeneration in the retina by activation of the inflammasome (90, 93, 94). Complex regulators are inhibited during retinal pathologies and this increases vascular alterations, dysfunction and photoreceptor cell death (95, 96). Indeed, there is clinical evidence that neuronal cell death in DR precedes vascular changes in the retina (97).

## Breakdown of the Blood-Retina Barrier in Diabetic Retinopathy

The BRB represents a functional separation of the circulatory system from the CNS, and it is sectioned into internal and external parts (98). The internal part comprises of a firmly linked capillary endothelial cell (EC) network covered by a stratum of pericytes and Müller cells that nourish the inner two-thirds of the retina whereas the outer part comprises of tightly joined pigment epithelial cells, whose role is to preserve the structural integrity of the outer retina layer (98). Pericytes play fundamental roles in a variety of processes, including angiogenesis, formation, and maintenance of the BRB, especially *via* their recruitment to growing retinal capillaries (99). The recruitment of pericytes to the sprouting retinal vasculature *via* regulated platelet-derived growth factor PDGF-B receptor beta (PDGFRβ) signaling is important for the complete formation of the functional BRB (100). Pericyte depletion is a major hallmark in the development of DR and it initiates BRB breakdown and subsequent formation of microaneurysms, edema, leakage, and ischemia (99).

Diabetes-induced glutamate release into the extracellular space causes neurodegeneration as seen in the db/db mouse (101). In a hyperglycemic environment, several mediators of inflammation contribute to the breakdown of the BRB. In STZ-diabetic mice, the retinal pigment epithelium (RPE) secretes VEGF which in turn triggers the recruitment of microglia to the RPE (102). Concurrently, microglia-derived TNF-α decreases protein levels of zonula occludens (ZO-1) in RPE cells, thereby disrupting the outer BRB in DR (102). Likewise, BRB breakdown was prevented in TNF-α knockout rats after six months of STZ induced diabetes implying that TNFα plays an important role in BRB disruption (103). The inhibition of colony stimulating factor 1 receptor (CSF1R) by PLX5622 leading to the ablation of microglia, reduced cytokine secretion in the retina and prevented BRB breakdown which confirms that microglia are drivers of BRB breakdown (104). Upon the breakdown of the BRB, the retina is filled with cytokines, chemokines, circulating proteins, infiltrating immune cells such as neutrophils and monocyte derived macrophages which together with overly reactive microglia advance the pathogenesis of DR. In this state, microglia reactivity is not only triggered by DAMPs of retinal origin but also from the circulatory system (44). The recruitment of blood-derived monocytes to the retinal tissue is a highly coordinated process occurring through CCL2-CCR2 signaling (105) and involves resident microglia (34). These dysregulated immune responses in the retina increase production of cytokines and chemokines which damage the retinal vasculature and neurons and causes DME and PDR.

## Innovative Animal Models of Diabetic Retinopathy

Several rodent models have been developed, either by induction (diet, drugs, laser, chemical damage) or genetic mutation (breeding) to investigate the pathophysiology of DR, and to design and assess novel therapies for DR. Disease phenotypes in STZ diabetic mice and rats include increased astrocyte gliosis, thinning of inner and outer retinal nuclear layers, pericyte ghosts, BRB breakdown, acellular capillaries, and basement membrane thickening (106). Genetic mouse models of DR differ in disease etiology and include Ins2^Akita^, non-obese diabetic (NOD), db/db (Lepr^db^), Kimba, and Akimba. The Ins2^Akita^ and NOD mice were developed to study type 1 diabetes whereas the db/db (Lepr^db^) mice were developed as models for type 2 diabetes. The Akimba mouse was generated by crossing the non-diabetic Kimba mouse model of proliferative retinopathy with diabetic Ins2^Akita^ mouse (107). Single cell RNA sequencing of Akimba mouse retinas has uncovered microglia as an immune cell cluster involved in the pathogenesis of DR (108). Although the mouse models of pharmacologically induced diabetes have contributed to our understanding of the morphological changes and the secretome of activated microglia cells, they fail to illustrate the neuronal and vascular changes associated with the development of DR in humans. As a result, the molecular and cellular mechanisms underlying DR pathogenesis once the BRB is compromised remains largely elusive.

This has necessitated the development of novel mouse models of DR that recapitulate the disease in human patients. For instance, the OIR mouse exhibits pathological neovascularization and nonperfusion, coupled with microaneurysm formation which reflect some aspects of DR (109). Binet and colleagues documented that microglia displayed a secretory phenotype during the peak of neovascularization at P17 in the OIR mouse (109). The db/db mouse also recapitulates neuroinflammatory features of human type 2 diabetes (101, 110). In these animals, the initially anti-inflammatory phenotype of microglia was overridden by pro-inflammatory cells during late stage DR (49). Several features characteristic of DR including vascular permeability, pericyte loss, BRB alteration, sustained hemorrhage, intra and sub-retinal fluid accumulation, retinal collapse, and blindness were reported in postnatal day 10 mice treated with an anti-platelet derived growth factor receptor beta (PDGFRβ) monoclonal antibody (APB5) (111). Administration of a single intraperitoneal injection of anti-PDGFRβ mAb at postnatal day 1 transiently blocked pericyte recruitment to the growing retinal capillaries and could reproduce several vascular hallmarks of DR in adult mice. Similarly, the *Pdgfb*^iΔEC^ mouse model generated by deleting platelet-derived growth factor (PDGF)-B in retinal endothelial cells (ECs) reproduced the key features of DR (99). These two mouse models offer excellent tools for studying the molecular and cellular processes mediated by activated microglia in the onset and development of DR.

## The Relevance of Modulating Microglial Reactivity in Diabetic Retinopathy

Given the clinical importance of inflammation in diabetic complications, modulation of microglia reactivity can be considered as a promising strategy in the management of DME and DR. The approach entails administering protective factors that mitigate the pro-inflammatory responses linked to DR. Several compounds including curcumin, quercetin and galangin are potent microglia modulators and their use improved disease outcome in murine DR models (112). Furthermore, minocycline which is a semi-synthetic tetracycline analog exhibits anti-inflammatory, and anti-apoptotic properties on microglia by suppressing the expression of TNF-α, IL-1β, NO, cyclooxygenase, and prostaglandins (113). In a retinal light damage study, minocycline counter-regulated microgliosis with concomitant preservation of the retinal structure (114). Minocycline also significantly reduced the number of amoeboid microglia in the outer retina and suppressed the expression of translocator protein 18kDa (TSPO) (114). Notably, reactive microglia express TSPO which modulates microglial inflammatory response, phagocytosis, and secretion of reactive oxygen species that can enhance retinal angiogenesis (115, 116).

## Conclusion

Microglia reactivity and elevated levels of pro-inflammatory cytokines and chemokines are important hallmarks in DR pathologies. Even though the precise molecular link between microglia and disease progression is unknown, studies have demonstrated that modulation of microglia can mitigate vision deficits. These findings imply that novel therapies that alleviate harmful microglia reactivity are of clinical importance in the future management of DR. Further studies in innovative mouse models of DR will help to advise scientists on the best therapeutic strategies towards combating DME and DR.

## Author Contributions

All authors contributed to the article and approved the submitted version.

## Funding

This work was supported by the German Research Foundation (DFG) research unit FOR2240 (project 6) and the CMMC Cologne (project B05).

## Conflict of Interest

The authors declare that the research was conducted in the absence of any commercial or financial relationships that could be construed as a potential conflict of interest.
